# Ibrutinib Treatment through Nasogastric Tube in a Comatose Patient with Central Nervous System Localization of Mantle Cell Lymphoma

**DOI:** 10.1155/2018/5761627

**Published:** 2018-08-16

**Authors:** Tamim Alsuliman, Maifa Belghoul, Bachra Choufi

**Affiliations:** ^1^Service d'Hématologie, Centre Hospitalier de Boulogne-sur-Mer, Boulogne-sur-Mer, France; ^2^Service d'Hématologie, Centre Hospitalier Régionale Universitaire de Lille, Lille, France; ^3^Augsburg College, Minneapolis, MN, USA

## Abstract

Nowadays, mantle cell lymphoma is considered to have one of the worst prognostic profiles among lymphoid malignancies. Mantle cell lymphoma rarely affects the central nervous system (CNS) as it represents about 0.9% of diagnosis and 4% among recurrent cases. Here, we present the case of a 69-year-old male patient who was diagnosed with mantle cell lymphoma in 2006. The patient relapsed three times, without affecting the CNS, then was treated accordingly, and achieved complete remission three times. Four years after his last complete remission, upon receiving his last dose of treatment, the medical team noted a rapid worsening of the patient's neurological status followed by a deep coma state causing MCL neurological recurrence by exclusion diagnosis. The patient then received ibrutinib via a nasogastric tube at a dose of 560 mg daily. Two days after receiving his last dose of ibrutinib, the patient regained full consciousness, and 10 days later, he was discharged from the hospital. The patient achieved complete remission and showed no signs of neurological damages for 24 months following his ibrutinib treatment. We believe that the administration of ibrutinib through the nasogastric tube was a determinant factor in this patient's remission and survival.

## 1. Introduction

Nowadays, mantle cell lymphoma is considered to have one of the worst prognostic profiles among lymphoid malignancies [1]. Its treatment is not widely uniformed [2]. High-dose aracytine, intensive chemotherapy followed by stem cells reinjection, rituximab maintenance, and more recently Bruton's tyrosine kinase (BTK) inhibitors are often the most frequently used elements in the therapeutic arsenal [3].

Mantle cell lymphoma (MCL) rarely affects the central nervous system (CNS). Indeed, the occurrence of mantle cell lymphoma in the CNS represents about 0.9% of all diagnosis of mantle cell lymphoma and 4% of recurrent cases [4].This type of localization is typically observed in high proliferating forms of MCL. CNS localization can involve many serious complications such as loss of consciousness [5]. There is no consensus on its treatment, and its survival is about 3–5 months after diagnosis [6]. Additionally, the efficacy of CNS prophylaxis, which typically includes aracytine and autologous stem cell transplantation, is yet to be proven. [7].

## 2. Patient and Method

This work was conducted with respect to the Helsinki declaration. Informed consent for the publication of this article was obtained from the patient.

This is a case of a 69-year-old male diagnosed with mantle cell lymphoma.

Ibrutinib was used at a daily dose of 560 mg, using a nasogastric standard feeding tube.

## 3. The Case

A 69-year-old male was diagnosed in 2006 with classical mantle cell lymphoma, Ki67 positive (cells positivity was close to 30%), involving the lymph nodes and the bone marrow. The patient was treated initially by 8 cycles of R-CHOP (rituximab 375 mg/m2, cyclophosphamide 750 mg/m2, doxorubicin 50 mg/m2, and vincristine 1,4 mg/m2 for a maximum dose of 2 mg), leading to a complete remission, and this treatment was applied following our local institutional guidelines for alternative treatments due to patient's refusal of ASCT at the moment.

Two years later, the patient had his first relapse with a nodal localization. The patient was treated with rituximab and high-dose aracytine-based immune-chemotherapy regimen (R-DHAP: rituximab, dexamethasone, cytarabine, and cisplatin) followed by autologous stem cell transplantation leading to a second complete remission.

Two years later, the patient was diagnosed with recurrent mantle cell lymphoma, treated this time with six cycles of rituximab and bendamustine combination therapy, and ended up in complete remission for the third time.

By August 2015, nearly 4 years after this third line of treatment, a significant right axillary nodal mass measuring 5 × 7 cm at its largest axes was discovered during his routine follow-up exam. A surgical biopsy and a pathological examination were carried out revealing MCL recurrence, with the same histological characteristics, and ibrutinib as a salvage therapy was prescribed shortly afterwards.

However, the patient was admitted in the neurology department due to a suspicion of cerebrovascular accident just prior to treatment initiation. A complete neurological exam was negative, and the patient was discharged with a prescription of aspirin. Fifteen days after his discharge from the hospital, the patient was admitted again secondary to a sudden onset of vertigo associated with a progressive loss of consciousness. Magnetic resonance imaging and a lumbar puncture revealed no obvious abnormalities. Extensive neurological and cardiovascular investigations were launched with no obvious pathology. Given the rapid degradation of the patient's neurological status followed by a deep coma state during his hospitalization, we decided to initiate ibrutinib treatment via the nasogastric tube at a daily dose of 560 mg. Two days later, the patient regained full consciousness, and 10 days later, he was discharged from the hospital.

Additional workup during the patient's follow-up appointments rejected all the other hypotheses (cerebrovascular accident, neuromeningitis, and focal lesions) behind the patient's neurological situation.

The patient was in complete remission after receiving ibrutinib and had no signs of neurological involvement for 24 months after the initiation of ibrutinib. However, after these two years, he developed a recurrent disease, with the same pathology (MCL), and a nodal localization. Currently, he is under treatment with RiBVD regimen (rituximab, bendamustine, Velcade, and dexametasone).

## 4. Discussion

To the best of our knowledge, this is the first case published describing the administration of ibrutinib through a nasogastric tube to a comatose patient. It is worth noting, however, that one case was reported describing the administration of ibrutinib capsule content through the nasogastric tube in a conscious patient with a swallowing disorder [8].

Although the manufacturer has already declared that this method of administration is not recommended, we found one on-going clinical trial comparing suspension and sprinkle formulations of ibrutinib to the capsule formulation (NCT02390609). However, the results for this study have not been released yet [9]. We believed, regarding the critical situation, that this mode of delivery of the drug could represent the last chance for this patient.

Bruton's tyrosine kinase is an essential component of B-cell receptor signalling which mediates the interaction between the tumor microenvironment and promotes the survival and proliferation of B-cells. Ibrutinib is the Bruton tyrosine kinase (BTK) inhibitor that acts against B-cell malignancies promoting inhibition of proliferation-inducing apoptosis [10]. Central nervous system (CNS) dissemination occurs in 4.1% of patients with mantel cell lymphoma, and median survival is of average 3 months [4, 11].

CNS localization of mantle cell lymphoma is a dramatic incident, and without ibrutinib, we believe that the patient would have died. Sophie Bernard and Catherine Thieblemont are among the first to have reported their experience with the use of ibrutinib to treat CNS in MCL patients with CNS relapse, showing rapid positive clinical response within 3 to 8 days, with certain patients having a higher ibrutinib plasma exposure on day 8 (AUC 0–24 h 3119 ng*∗*h/mL; C_max_ 729 ng*∗*mL^−1^). Ibrutinib concentration was measured in the cerebral spinal fluid to be 1 to 7 percent of plasma concentration, assuming that ibrutinib had reached CSF, though neither the permeability nor the diffusion of ibrutinib through the blood-brain barrier have been well studied. Yet, the mechanism of ibrutinib CSF distribution and its target are still not well understood.

In the aforementioned case series, the survival ranged from some months to a year after CNS relapse diagnosis, and the responses were active at 6 to 12 months of follow-up for two confirmed complete remissions, while a partial response was obtained for the third. In addition, these findings may be discussed along with ibrutinib treatment results of similar situations, as primary CNS lymphoma [12]. Obviously, we could not totally roll out the occurrence of encephalitis mediated by paraneoplastic anti-neuronal antibodies, though the absolute negativity of MRI repeated examinations for any habitual sign of paraneoplastic encephalitis along with the rapid and total resolving of all neurological symptoms after 2 days of ibrutinib initiation and the long MCL history are strongly indicating MCL neurological involvement as the main hypothesis [13].

## 5. Conclusion

In light of the aforementioned arguments, we believe that the treatment by ibrutinib through the nasogastric tube was a determinant factor in this patient's survival. However, more studies should be conducted to evaluate the efficacy and safety of this molecule in CNS localization of mantle cell lymphoma and maybe even primary CNS lymphoma.
